# Case Report: Aveir implantation in an 8.7-year-old, 25-kg pediatric patient with mitochondrial disease via internal jugular vein

**DOI:** 10.3389/fcvm.2025.1593438

**Published:** 2025-10-10

**Authors:** Xue Zhou, Xin Xu, Shuang He, Qian Liu, Tiewei Lu

**Affiliations:** ^1^ Department of Cardiovascular Medicine, Children’s Hospital of Chongqing Medical University, Chongqing, China; ^2^ National Clinical Research Center for Child Health and Disorders, Chongqing, China; ^3^ Ministry of Education Key Laboratory of Child Development and Disorders, Chongqing, China; ^4^ Chongqing Key Laboratory of Pediatrics, Chongqing, China; ^5^ Key Laboratory of Children's Important Organ Development and Diseases of Chongqing Municipal Health Commission, Chongqing, China; ^6^ National Clinical Key Cardiovascular Specialty, Chongqing, China

**Keywords:** Aveir, pacing, transjugular venous, pediatric, mitochondrial disease

## Abstract

**Introduction:**

Mitochondrial disorders frequently precipitate progressive conduction system degeneration, with complete heart block representing a critical therapeutic challenge in pediatric populations, which ultimately requires permanent pacemaker implantation. However transjugular venous implantation of the Aveir leadless pacemaker in a pediatric patient with mitochondrial disease has not been previously reported.

**Methods and results:**

An 8.7-year-old, female patient (weight: 25 kg) with genetically confirmed mitochondrial disease presented with cardiac syncope secondary to the complete atrioventricular block. Comorbidities included insulin-dependent diabetes and pocket infection from a conventional permanent pacemaker implanted four months prior. Given her history of device-related complications and restricted venous access, a retrievable leadless pacemaker (Abbott Aveir) was successfully implanted via the internal jugular vein under fluoroscopic guidance without complication. Stability testing confirmed adequate fixation with pacing threshold of 1.0V@0.2 ms and impedance of 610Ω at one-month follow-up.

**Conclusion:**

The Aveir VR leadless pacemaker can be safely implanted via the internal jugular vein approach in pediatric patients with mitochondrial disease.

## Introduction

Mitochondrial diseases commonly manifest with progressive cardiac conduction system disorders, often presenting as symptomatic bradycardia that ultimately requires permanent pacemaker implantation (1). Traditional transvenous permanent pacemaker implantation carries significant risks, including device- and pocket-related infections, as well as lead-associated complications. These risks are particularly elevated in pediatric populations due to their smaller anatomical dimensions and limited endovascular space, resulting in an elevated incidence of implantation-related complications. Furthermore, mitochondrial disorders often involve systemic comorbidities such as diabetes mellitus (DM), which further increases postoperative risks. Although leadless pacemakers have substantially mitigated these challenges and gained widespread acceptance with proven utility in adult populations (2, 3), their application remains limited in pediatric patients, particularly those with complex comorbidities like mitochondrial diseases and DM.

We report the first documented successful implantation of an Aveir VR leadless pacemaker via right internal jugular vein access in a pediatric patient with mitochondrial disease, DM, symptomatic complete atrioventricular block, and prior pacemaker pocket infection. This study was approved by the Ethics Committee of Children's Hospital of Chongqing Medical University, with written informed consent obtained.

## Case presentation

**Figure 1:**
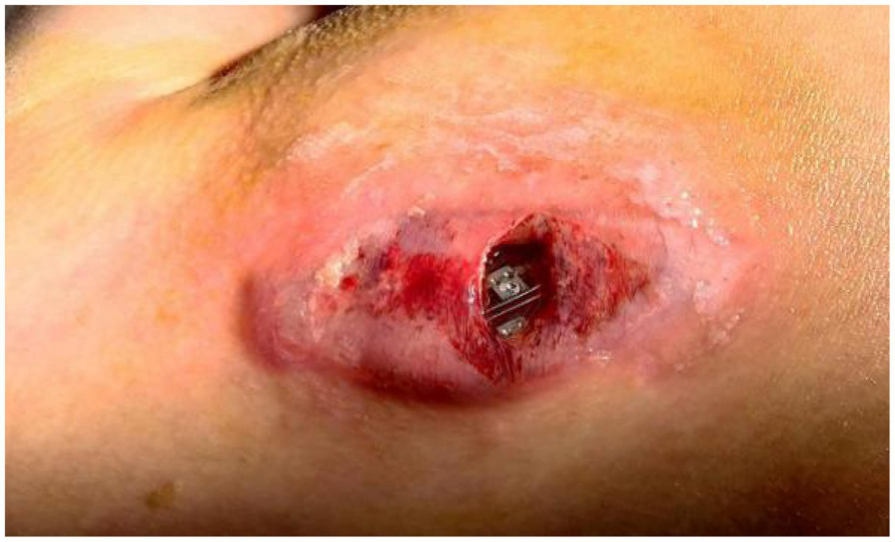
A device-related pocket infection manifested four months after transvenous pacemaker system implantation.

**Figure 2:**
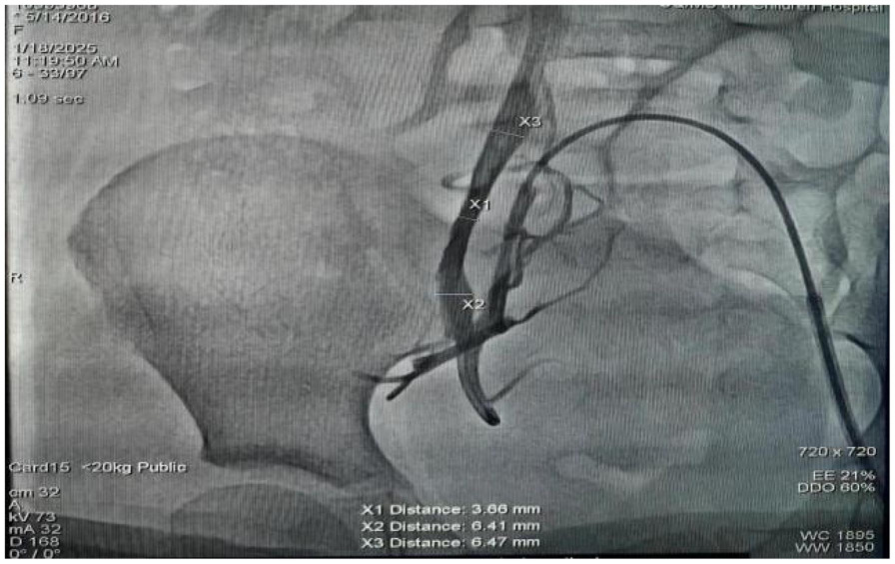
Right femoral venography revealed a smallest diameter of 3.66 mm in the right femoral vein.

An 8.7-year-old female patient (weight: 25 kg) was admitted to our cardiovascular department with symptomatic pacemaker pocket infection. Her medical history included DM, retinal pigment abnormalities, progressive atrioventricular block culminating in a diagnosis of mitochondrial disease confirmed by mitochondrial DNA deletions. She had previously experienced cardiac syncope secondary to the complete atrioventricular block, necessitating conventional permanent pacemaker implantation. Post-implantation device testing confirmed appropriate pacemaker function with 100% right ventricular pacing. However, four months post-implantation, she developed a pacemaker pocket infection (Figure 1). Given her DM and the associated increased risk of recurrent infection, the entire pacemaker system was explanted. Permanent pacing remained clinically indicated due to progressive symptomatic atrioventricular block in the context of mitochondrial disease. Although re-implantation of a conventional transvenous pacemaker still carried significant infection risk, a leadless pacemaker offered a safer alternative with reduced infection potential. Preprocedural right femoral venography revealed a minimum vein diameter of 3.66 mm (Figure 2), which was insufficient for the 27-Fr introducer sheath required for femoral approach. However, the right internal jugular (RIJ) vein, with a minimum diameter of 6 mm, was deemed suitable for device implantation. After discussing the options for transvenous vs. leadless pacing system and alternative venous access sites with family, we proceeded with Aveir VR leadless pacemaker implantation via the RIJ approach.

## Methods

**Figure 3:**
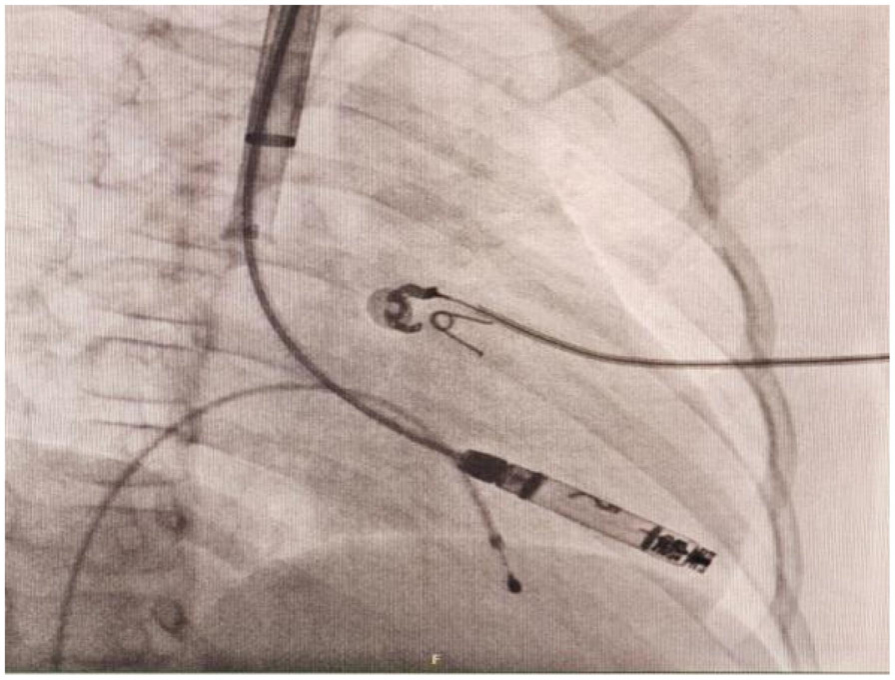
Right anterior oblique (RAO) projection confirmed the Aveir VR leadless pacemaker was advanced to the right ventricle through right internal jugular vein.

Following successful induction of anesthesia, ultrasound-guided RIJ vein puncture was performed with placement of a 6-Fr sheath. Following venography, a vascular closure device was preplaced (Perclose ProGlide, Abbott). A pigtail catheter was then advanced over a guidewire into the right ventricle via the RIJ vein. Angiography was performed in RAO 30°/0° and LAO 45°/0° projections to delineate the right ventricular anatomy and identify optimal apical/septal pacing sites. The pigtail catheter was then exchanged for an Amplatz Super Stiff guidewire (0.035 cm, 260 cm), allowing sequential venous dilation from 8-Fr to 24-Fr. The 27-Fr Aveir introducer sheath (outer diameter) was advanced to the SVC- right atrial junction after thorough flushing. The tapered dilator was then carefully removed, and the sheath was connected to heparinized saline infusion. Subsequently the Aveir delivery catheter (25-Fr) was introduced through the 27-Fr outer sheath. Under fluoroscopy, the delivery system was navigated across the tricuspid valve to an apical-RV septal position. Angiography confirmed optimal device placement and septal position (Figure 3), with mapping demonstrating an R-wave amplitude of 4.0 milliVolts (mV), impedance of 330Ω, pacing threshold of 1.75V@0.4 milliseconds (ms), injury current of 1.5 mV. The Aveir leadless pacemaker was deployed in the RV apical septum with 1.4 clockwise rotations under fluoroscopic observation. Subsequent deflection testing confirmed stable fixation. Post-deployment electrical measurements showed improved pacing performance with pacing threshold of 0.5V@0.4 ms, R-wave amplitude of 3.8 mV, impedance of 490Ω. The leadless pacemaker was released after final programming to VVI mode at 55 beats per minute (bpm). Catheters were withdrawn under fluoroscopic guidance, and hemostasis was achieved using the pre-deployed PerClose vascular closure device combined with subcutaneous suturing. The patient maintained hemodynamic stability throughout the procedure without complications. Intraoperative programming data for the leadless pacemaker are detailed in Table 1. At one-day follow-up, the device demonstrated a pacing threshold of 0.75V@0.2 ms, impedance of 510Ω, R-wave amplitude of 6.7 mV and predicted longevity of 15.4 years (with 100% pacing). Transthoracic echocardiography confirmed apical-septal position without tricuspid regurgitation or pericardial effusion. At one-month follow up, the catheter insertion site was well-healed, with a pacing threshold of 1.0V@0.2 ms, impedance of 610Ω. The device's predicted longevity was 14.3 years. During follow-up, the child exhibited complete resolution of syncope and resumed age-appropriate activities. This clinical outcome provided substantial reassurance to the patient and parents regarding long-term management.

**Table 1:** Intraoperative programming data for the leadless pacemaker.

Programming	Damage current	Impedance	Threshold	Pulse width	Sensing
Positioning test before screw-in	1.5 mV	330Ω	1.75 V	0.4 ms	4.0 mV
Screw-in for 1.0 turn	2.5 mV	460Ω	0.5 V	0.4 ms	–
Screw-in for 1.4 turns	3.0 mV	510Ω	–	0.4 ms	4.0 mV
Deflection test	3.2 mV	510Ω	0.5 V	0.4 ms	–
Release pacemaker	2.5 mV	490Ω	0.5 V	0.4 ms	3.8 mV

## Discussion

Although transjugular leadless pacemaker implantation demonstrates established safety and efficacy in pediatric patients under 30 kg (4), clinical evidence specific to the Aveir VR system remains limited. This evidence gap is particularly pronounced in pediatric populations. We report the successful implantation of an Aveir VR leadless pacemaker in an 8.7-year-old pediatric patient with complex comorbidities. To our knowledge, this represents the first documented implantation of an Aveir VR leadless pacemaker via right internal jugular vein access in a child with coexisting mitochondrial disease, DM, and prior pacemaker pocket infection necessitating complete system explantation. This alternative vascular access approach offers significant clinical implications for managing pediatric pacing candidates with anatomical constraints or infection risk.

Cardiac manifestations pose a significant clinical challenge in mitochondrial disorders, with cardiomyopathy and conduction system abnormalities being particularly prevalent. The prevalence of conduction system disease in mtDNA diseases shows an age-dependent increase in the general population. Consequently, the following management priorities are essential: (1) Vigilant cardiac monitoring: Close surveillance for the progressive nature of conduction disease and sudden cardiac death (SCD) risk is paramount, an aggressive prophylactic pacing strategy may be indicated to prevent SCD (5). (2) Comprehensive cardiomyopathy assessment: Concomitant myocardial disease must be rigorously evaluated, preferential use of physiological pacing (His-bundle pacing or left bundle branch area pacing) may be the preferred modalities when feasible and appropriate. When physiological pacing is not employed, device programming should actively minimize unnecessary right ventricular pacing to preserve cardiac function. (3) Strategic device system selection: Active-fixation leads and MRI-conditional systems are imperative, individualized long-term programming and surveillance protocols are essential.

In this pediatric case, progressive atrioventricular block evolved to complete heart block with syncope, complicated by pacemaker pocket infection requiring complete system explantation. This presentation met Class I indications for permanent pacemaker implantation (1). The decision between transvenous and leadless pacing for reimplantation requires careful consideration of several unique factors: the risk of device-related infection varies significantly between initial implantation and subsequent interventions, with reported rates of approximately 0.5% for primary procedures, and 1%–7% for secondary procedures including generator changes, lead modifications, and system upgrades (6). Patients with mitochondrial disease exhibit compromised myocardial energetics and frequent endocrine comorbidities like DM, predisposing to infectious complications. Additionally, traditional transvenous pacemaker implantations also carry inherent risks of mechanical myocardial injury during lead fixation (7). And the ability to grow, limited vascular access, and inevitable requirement for periodic hardware upgrades in pediatric cases necessitate modifications in both lead selection and implantation techniques (8, 9). Given these constraints, leadless pacemaker technology offers a safe and effective alternative for this population, providing dual benefits of reduced infection risk and elimination of lead-related complications.

Leadless technology offers significant advantages by avoiding long-term complications associated with transvenous leads, such as lead fracture (1%–4%) (10, 11), moderate-to-severe tricuspid regurgitation (5%) (12, 13) and infection (1%–2%) (14, 15). Recently, the Abbott Aveir VR leadless pacemaker, which was approved for clinical use in 2022, has seen increasing clinical adoption. Its helix-fixation design enables retrieval capability and comprehensive pre-deployment electrical parameter assessment, potentially reducing post-deployment re-positioning needs (16). Additionally, the Aveir VR has a larger battery capacity compared to Micra (243 mAh vs. 120 mAh) at equivalent pacing parameters, which could potentially extend device longevity and decrease the likelihood of premature system replacement. However, careful patient selection remains critical, particularly in pediatric population, due to challenges such as small body size, underdeveloped vasculature, limited venous access, and small cardiac chamber dimensions.

While femoral vein access remains the standard implantation route of Aveir VR, emerging evidence merits consideration of alternative approaches, particularly when femoral access is contraindicated or undesirable. Although current literature primarily describes internal jugular vein access in isolated case reports, this technique is supported by established safety profile of Micra implants via jugular access (17–19). In this case, the right femoral vein's critical luminal diameter (3.66 mm) was insufficient to accommodate the 27-Fr introducer sheath (25-Fr inner diameter), precluding femoral delivery. Consequently, the device was successfully deployed via an internal jugular venous approach following comprehensive preprocedural vascular mapping and real-time ultrasound-guided catheter navigation. The internal jugular venous approach offers an anatomically favorable trajectory for right atrial access due to its superoanterior orientation, which significantly shortens the intracardiac catheter pathway. This route eliminates navigation of the acute IVC-tricuspid angle while optimizing coaxial alignment with the ventricular septum for targeted deployment. Clinical data show significantly reduced mean procedure time, fluoroscopy time, and radiation dose compared to femoral access (14). However the movements to enter the right ventricular from the superior approach are opposite of those from the femoral vein, which may increase the challenge of achieving a safe implant and require meticulous torque control to counterbalance the inherent clockwise rotational forces during right ventricular engagement.

## Conclusion

Aveir VR leadless pacemaker can be safely implanted via the internal jugular vein approach in pediatric patients with complex comorbidities. It represents a paradigm shift in pediatric pacing, offering a promising alternative to traditional transvenous systems in high-risk patients.

## Data Availability

The raw data supporting the conclusions of this article will be made available by the authors, without undue reservation.
